# Modeling of Beta Diversity in Tunisian Waters: Predictions Using Generalized Dissimilarity Modeling and Bioregionalisation Using Fuzzy Clustering

**DOI:** 10.1371/journal.pone.0131728

**Published:** 2015-07-06

**Authors:** Frida Ben Rais Lasram, Tarek Hattab, Ghassen Halouani, Mohamed Salah Romdhane, François Le Loc'h

**Affiliations:** 1 Unité de Recherche UR03AGRO1 Ecosystèmes et Ressources Aquatiques, Institut National Agronomique de Tunisie, Tunis, Tunisia; 2 Unité de Recherche Ecologie et Dynamique des Systèmes Anthropisés (EDYSAN, FRE 3498 CNRS-UPJV), Université de Picardie Jules Verne, Amiens, France; 3 Laboratoire des Sciences de l'Environnement Marin UMR 6539 LEMAR (CNRS/UBO/IRD/Ifremer), Institut Universitaire Européen de la Mer, Technopôle Brest-Iroise, Plouzané, France; Università di Genova, ITALY

## Abstract

Spatial patterns of beta diversity are a major focus of ecology. They can be especially valuable in conservation planning. In this study, we used a generalized dissimilarity modeling approach to analyze and predict the spatial patterns of beta diversity for commercially exploited, demersal marine species assemblages along the Tunisian coasts. For this study, we used a presence/absence dataset which included information on 174 species (invertebrates and fishes) and 9 environmental variables. We first performed the modeling analyses and assessed beta diversity using the turnover component of the Jaccard’s dissimilarity index. We then performed nonmetric multidimensional scaling to map predicted beta diversity. To delineate the biogeographical regions, we used fuzzy cluster analysis. Finally, we also identified a set of indicator species which characterized the species assemblages in each identified biogeographical region. The predicted beta diversity map revealed two patterns: an inshore-offshore gradient and a south-north latitudinal gradient. Three biogeographical regions were identified and 14 indicator species. These results constitute a first contribution of the bioregionalisation of the Tunisian waters and highlight the issues associated with current fisheries management zones and conservation strategies. Results could be useful to follow an Ecosystem Based Management approach by proposing an objective spatial partitioning of the Tunisian waters. This partitioning could be used to prioritize the adjustment of the actual fisheries management entities, identify current data gaps, inform future scientific surveys and improve current MPA network.

## Introduction

Human well-being is intrinsically linked to biodiversity (e.g. [1]), a well-recognized fact driving the increasing interest in the effective preservation of ecosystems. One of the fundamental paradigms underpinning these conservation efforts is ecosystem-based management (EBM) [2], an approach which considers the whole ecosystem (including humans), instead of managing a single species or threat in isolation. Implementing EBM requires significant spatial information, such as distribution information on species, assemblages or communities [3,4,5]. To determine spatial variations at a local scale, many current management programs use an alpha diversity modeling approach. Alpha diversity can be measured by either using species richness values or diversity indices (e.g., the Shannon-Weiner index or the Simpson index). Gamma diversity is often used at regional scales and quantifies total species diversity across a group of local scale habitats or sites [6]. However, the foundations underlying marine species diversity distribution are more complex than the local or the regional diversity. Indeed, the diversity of a region is determined more by differences in biological composition between locations (i.e. beta diversity) than by site-level diversity [7]). Hence, it is more challenging to base conservation assessments using beta diversity than either alpha or gamma diversity.

The concept of beta diversity was first introduced by Whittaker (1962, 1970) [8–9] to define the extent of differentiation that existed in communities along a habitat gradient. In other words, beta diversity explains the variations in species composition that can occur among individual sites within the same geographic area. Whittaker (1970) also proposed that beta diversity could be measured as the ratio between gamma and alpha diversity. Since this first work, many alternative approaches to measuring beta diversity have been suggested, including the Jaccard or Sørensen dissimilarity indices, which measure dissimilarity in species assemblages [10], the slope of a linearized species-accumulation curve [11], and the parameter of a power function [12]. To date, no consensus has been reached regarding the reliability of the different methods; Koleff *et al*. (2003) [10] showed that each approach leads to a different assessment of beta diversity. The most popular approach is to use a dissimilarity index [13–14]. Ultimately, whichever method is selected, it is certain that quantifying the spatial turnover of species (i.e., beta diversity) is critical for effectively delineating biotic regions and conservation planning [14–15].

Measuring beta diversity requires accurate and spatially-explicit species data. However, acquiring such data at the necessary spatial resolution can be costly and laborious. Moreover, data are mostly in coastal areas, in shallow part of the water column and this induces an important bias with low trophic level species that are generally non-exploited [16]. Thus, many ecosystems are only patchily surveyed. To overcome this issue, remotely mapped information can be used (e.g., habitat type, abiotic variables) to predict diversity as a function of environmental variables. This can be achieved using species distribution models or habitat suitability models allowing for extensive data extrapolation [17]. Generating predictions of beta diversity within unsurveyed areas across a region of interest is allowed by the generalized dissimilarity models (GDM). First proposed by Ferrier *et al*. (2007) [7], GDM depend on a statistical technique to analyze and predict spatial patterns of beta diversity that relates biological distance to distance in environmental space. This approach is an extension of matrix regression and is specifically designed to accommodate the nonlinearity commonly encountered in large-scale ecological datasets [7]. These models can be used to meet various conservation planning requirements, including bioregionalization, survey gap analyses and climate change impact assessments [7].

Although the evidence suggests that it is more challenging to base conservation assessments using beta diversity, relatively little attention has been given to the application of marine beta diversity values. Moreover, the use of GDMs to address the issues associated with patchy data have also been largely overlooked (e.g., [18–19]). In this study, we used GDM to analyze and predict spatial patterns of beta diversity for commercially-exploited demersal assemblages along the Tunisian coast. During the last decade, commercially-exploited demersal species represents nearly 48% of the total landings and contributes 68% of the fisheries products economic values in Tunisia [20]. Situated at the junction of the eastern and western Mediterranean basins, Tunisia’s marine ecosystems are particularly interesting. They are considered a biodiversity hotspot (support > 50% Mediterranean fish species; [21–22]), and exhibit diverse biotic and abiotic characteristics. The Tunisian exclusive economic zone (EEZ) is also considered to be one of the most anthropogenically-impacted areas in the Mediterranean Sea [23]. Specifically, we set out to (*i*) identify and delineate the biogeographical transition zones within the Tunisian EEZ by assessing beta diversity patterns and (*ii*) identify a set of indicator species for each biogeographical region that can be used to evaluate possible interdependencies between the broader species assemblages and current environmental conditions.

## Materials and Methods

### Study Site

**Fig 1:**
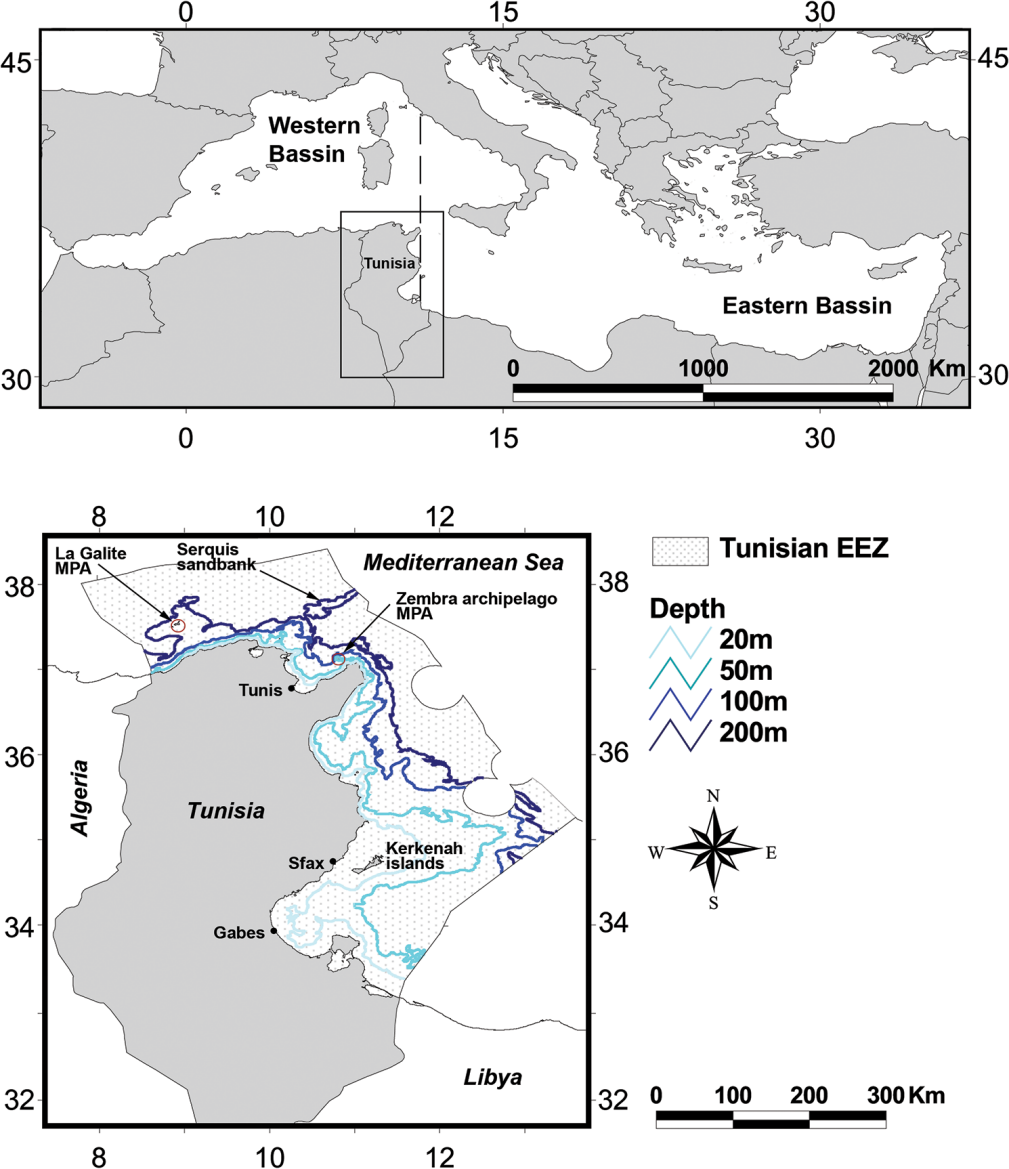
Geographical location of the study area and main geographical features of the Tunisian exclusive economic zone (EEZ). The axes indicate degrees latitude and longitude.

Tunisia is located in the southern Mediterranean Sea and lies in the transition zone between the eastern and western basins (Fig 1). Tunisia’s 1670 km coastline features a variety of demersal habitats: to the north, the seafloor is a mixture of rock and soft sediments; the continental shelf is narrow, the slope is steep and the biodiversity is high [24]. In contrast, the eastern coastline features an extended shelf and a less rocky seafloor; however, the biodiversity level is relatively similar to the northern region [24]. The Gulf of Gabes in southern Tunisia, which features the second widest continental shelf region in the Mediterranean Sea, is of particular economic and ecological interest. Its highly productive ecosystem supports significant fisheries activities [25] and the ecologically-important Posidonia seagrass meadows. A 2005 review of Tunisia’s marine biodiversity (including all taxons except plankton and mammals) revealed that the northern region supports 867 species, the eastern region 292 species, and the southern region 667 species [26]. In this study, we will focus on the entire EEZ, which encompasses an area of 101,809 km^2^ (Fig 1).

### Species data

Since 1998, the National Institute of Marine Sciences and Technologies (INSTM) has regularly monitored Tunisian marine biodiversity and made their data (presence/absence data) freely available through the Ocean Biogeographic Information System (OBIS; http://www.iobis.org/). We collated data on 174 species from OBIS (accessed on December 2014; 133 fishes, 18 cephalopods, 22 decapods and one stomatopod; S1 Table). For each species, we obtained trawl data, completed between 1998 and 2005, with central point georeferenced positions and the associated presence/absence information.

The INSTM dataset we used belongs to a standardized and validated database (see Hattab *et al*. (2013) [27]) for more details about the survey data protocol) that have been already used in previous studies (e.g. [27,28,29]). We proceeded to a data quality check by validating species names and searching for synonymy based on WoRMS database (WoRMS Editorial Board 2013 [30]).

To ensure the data represent the full annual turnover of species per year, we checked the monthly frequency of the samplings (S1 Fig).

### Environmental data

We selected nine variables to predict beta diversity that have previously been shown to strongly influence species distribution patterns in coastal environments [31,32] and particularly for local scale [27]. These included climate variables (sea surface temperature (SST) and sea surface salinity (SSS)), local-scale habitat variables (bathymetry, bathymetric slope and aspect) and spatial predictor (distance to shore). For SST (2002–2010; MODIS) and SSS (1955–2006; NOAA’s World Ocean Atlas), we used monthly climatology data to derive annual mean values. We also identified the annual range for each 12 month period using the maximum and minimum monthly values, a metric that can be used to represent seasonality. We utilize the full temporal extent of the climate datasets in making these climatologies to better reflect regional disparities.

As this study deals with demersal species, we tested if surface parameters show the same spatial distribution and temporal variation than seafloor parameters derived from MEDAR/MEDATLAS datasets (S2 Fig).

To derive the habitat and spatial predictor variables, we used publicly available raster data that we sourced from MARSPEC’s SRTM30_PLUS high resolution bathymetry dataset (www.marspec.org). To accurately describe aspect, we used two variables, the eastness and the northness of the slope [33]. Aspect represents the azimuthal direction of the steepest slope and was transformed into two derived variables: Eastness (values close to 1 represent an eastward aspect, while values close to –1 represent a westward aspect) and Northness (values close to 1 represent a northward aspect, while values close to –1 represent a southward aspect).

Overall, the nine predictor variables used to predict beta diversity are mean annual SST, annual range in SST, mean annual SSS, annual range in SSS, bathymetry, bathymetric slope, aspect (eastness), aspect (northness) and distance to shore.

### The Generalized Dissimilarity Modeling Approach

The GDM approach uses recorded species data from a range of locations across the study site to fit a model that predicts the compositional dissimilarity between pairs of locations as a nonlinear multivariate function of the environmental attributes of these locations [7]. It is a reformulation of the Mantel approach into a generalized linear model in which a single response matrix can be modeled as a function of distance matrices of a number of explanatory variables [7]. By using a GDM approach, we can overcome two major problems: non-linearity in community dissimilarity between sites and ecological distance and uneven rates of species turnover along environmental gradients [34]. A key strength of GDM is that it uses flexible splines (constrained to be positively monotonic [34]) instead of parametric transformations of the variables.

We developed our GDM using a downloadable toolkit package (‘Gdm01’) for R software. This package provides a matrix-regression tool that models compositional dissimilarity as a function of environmental dissimilarity and geographic distance. We selected the three I-spline basis function option [7,34] and accounted for spatial autocorrelation by including the geographic distance between pairs of sites as a predictor variable. To evaluate the impact of each environmental variable, we ran multiple GDMs and removed one predictor at a time. We then compared the variance of the full GDM to each of the partial models to evaluate the importance of environmental variables on the change of communities.

### Measuring beta diversity

Beta diversity has two components: species turnover and nestedness of assemblages. The first component measures the rate with which one set of species replaces another set from site to site. Nestedness quantifies differences in species richness between sites [35]. Several recent papers [13,36,37] have demonstrated the importance of considering only species turnover, while measuring the level of differentiation between species assemblages. Therefore, we do not consider nestedness. Indeed, if we had of taken this component into account, the Jaccard dissimilarity index used here would have underestimated the influence of turnover on total dissimilarity while overestimating the influence of richness differences on total dissimilarity. Thus, we quantified compositional beta diversity for all pairwise grid cell combinations using the turnover component of the Jaccard’s dissimilarity index (*β*_*jtu*_; [37]). This is expressed as:

$$\beta_{jtu}=\frac{2\min\left(B,C\right)}{A+2\min\left(B,C\right)}$$

where for each pair of grid cells, *A* is the number of species that exist at both sites *i* and *j*, *B* is the number of species found in cell *i* but not in cell *j* and *C* is the number of species found in cell *j* but not in cell *i*.

### Ordination and clustering

To identify the biogeographical transition zones, we performed a nonmetric multidimensional scaling (NMDS) neighbor-joining algorithm to plot the observed and predicted beta diversity distance matrix along reduced axes [13,38].

The observed *β*_*jtu*_ dissimilarity matrix has a dimension of 692 x 692 since the observed data belong to 692 sites. The predicted *β*_*jtu*_ dissimilarity matrix has a dimension of 16796 x 16796. This matrix is generated using environmental data derived form rasters containing 16796 pixels.

To reach a stable solution and avoid the local minima, we performed a two-dimensional NMDS with 100 random starts. Stress values (i.e., the sum of the squared differences between the fitted and original distances) were used to assess how well the configuration of the points in the reduced ordination space matched the original distance matrix [39]. These values ranged from 0 to 1, with lower values indicating a better fit [39]. The ordination results were then plotted and mapped by assigning a color to each grid cell according to its position in the ordination space [13].

The ability to classify communities is a central requirement to distinguish, name and synthesize them [13]. Community patterns can be determined using NMDS results, though transition zones between communities may be gradual, occurring in response to environmental gradients. Consequently, it is not uncommon for this approach to produce areas of ambiguity that display properties of more than one community. Hierarchical cluster analysis, an approach frequently used to classify regional faunas [13], produces singletons. This approach assumes that one internally homogenous community exists at each point of space, with all communities excluded to the same degree [40] and any ambiguity in classification viewed as an error. Fuzzy C means (FCM) is an alternative approach that actively recognizes the “fuzzy” properties of communities [41] to identify fuzzy clusters instead of discrete clusters [42]. The FCM is a form of the K means analysis, meaning it requires the number of groups to be predefined [43]. Here, we chose to fix this number to three, reflecting the current number of fisheries management zones in place thereby facilitating comparisons. A fuzziness parameter (*m*) of 2 was used, based on the optimal range of 1.5 to 3.0 [42]. Fuzzy clustering results were displayed as maps that displayed the degree of membership to each group. The final color of each fuzzy community [43] was determined using the primary color levels taken from the centroid of its corresponding cluster on the NMDS axes.

**Fig 2:**
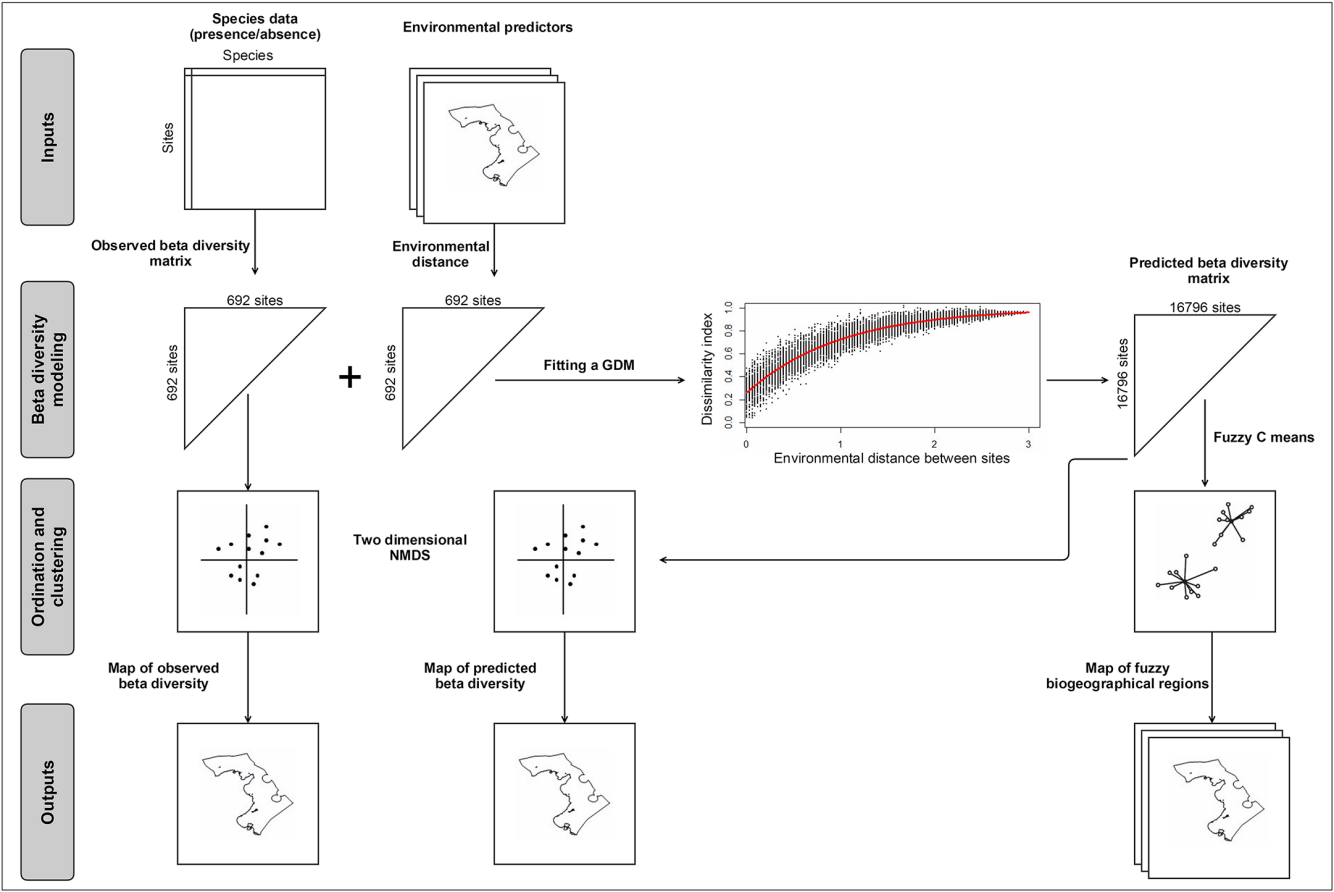
Modeling framework adopted in the present study.

The modeling framework with all the data processing steps is illustrated in Fig 2.

### Indicator species

We used the Dufrêne–Legendre indicator species analysis to identify indicators for each of the three biogeographical regions defined in this study [44]. Using this analysis, we calculated an indicator value (IndVal^g^) for each species considered. The IndVal^g^ is a modified version of the original Dufrêne–Legendre’s indicator value (IndVal), which allows the user to better control the effect of unequal site group sizes [45]. The IndVal^g^ metric is defined as the product of specificity and fidelity, where specificity is the probability that the surveyed site belongs to the target site group (on the basis that a target species has been found) and fidelity is the probability of finding the target species in sites belonging to the site group [45]. Indicator species were selected as those that had a significant IndVal^g^ that was greater than 0.6 (*p* < 0.001; significance was assessed by running a 9999 permutation test). This value was selected to ensure a reasonable number of species were selected for each region.

## Results

Our dataset included information on a total of 692 sites and 174 species from 77 families. The most represented families in this dataset were Sparidae (21 species), Rajidae (9 species) and Gadidae (6 species). On average, species were prevalent at 30% of sites. The most prevalent species occurred at 58% of the studied sites, while the least prevalent species only occurred at only 1% of the studied sites

### Generalized Dissimilarity Modeling

The GDM explained 63% of the variation in community composition. Following a comparison of the different GDM results, it was clear that aspect along the east-west axis was the most important explanatory variable for changes in demersal species composition (Table 1). This was followed by mean annual SSS and bathymetry (Table 1). Conversely, the annual SSS range and aspect along the north-south axis had no influence on community composition.

**Table 1:** Contributions of the variables for the GDM.

Variable	Dev _variable i_-Dev	%
Aspect (eastness)	124	17.7
Mean annual SSS	81	11.9
Bathymetry	36	5.1
Bathymetric slope	33	4.7
Distance to shore	20	2.9
Annual range in SST	15	2.2
Mean annual SST	13	1.9
Aspect (northness)	4	0.5
Annual range in SSS	0	0.1

Change in the deviance when the variable was removed from the model is shown, as well as the percentage contribution to the model (sea surface salinity (SSS), sea surface temperature (SST)).

### Spatial patterns of predicted beta diversity

**Fig 3:**
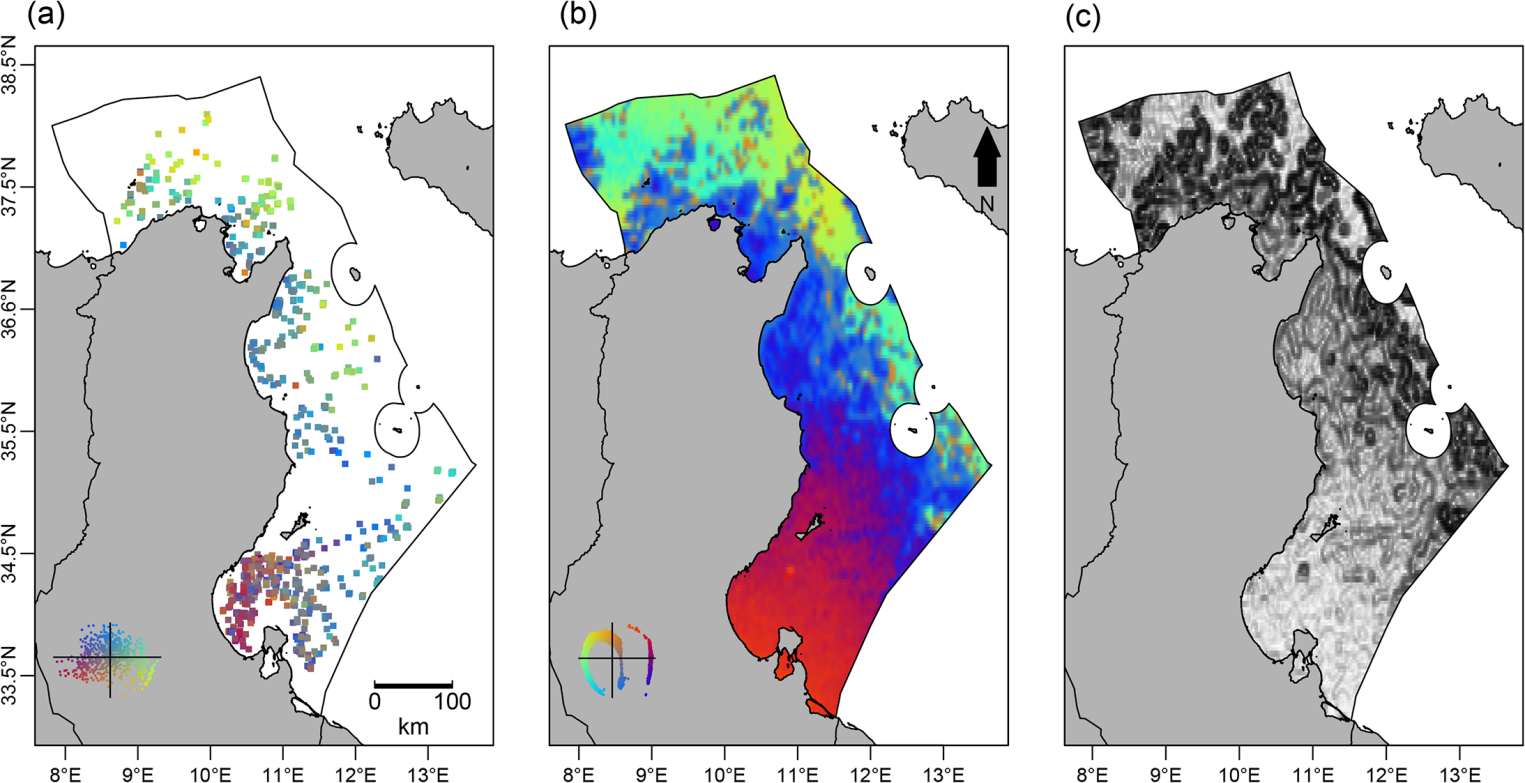
Observed (a) and GDM predicted (b) spatial patterns of beta diversity for demersal exploited marine species assemblages along the Tunisian coasts and the slope of the predicted beta diversity (c). Grid cells mapped in a similar color are predicted to have similar species assemblages, while cells mapped in a very different color are predicted to be highly dissimilar in composition.

The NMDS ordinations led to satisfactory projections of both the observed and predicted *β*_*jtu*_ dissimilarity matrices (with the dimensions 692 x 692 and 16796 x 16796, respectively) into a two-dimensional space. This was indicated by the relatively low stress values we obtained (0.19 and 0.20, respectively). The projection of the predicted *β*_*jtu*_ onto the NMDS axis showed a Guttmann effect, which means that the ordination axes are linked by a non-linear relationship and they describe a single ecological phenomenon [46]. The spatial beta diversity patterns were visualized using color maps, with similarly colored grid cells predicted to have similar species assemblages. Conversely, cells mapped in very different colors are predicted to have highly dissimilar species assemblages. The observed and GDM predicted beta diversity maps (Fig 3A and 3B) show similar spatial patterns: both exhibit an inshore-offshore gradient and a latitudinal gradient (from south to north).

The GDM map shows a distinct difference in species assemblages between coastal and offshore areas. For example, the shallow Gulf of Gabes supports coastal assemblages that differ from those found in the deeper regions. A south to north gradient is also distinguishable, e.g., the deeper waters found in the far northern region exhibited the highly distinct deep-sea assemblages. These were also encountered off the eastern Tunisian coast and in a small patch off the Gulf of Gabes where the continental slope begins. The coastal areas of northern and eastern Tunisia appear to have a similar species composition. It appears that the transition between the Gulf of Gabes and the eastern coastal areas is relatively soft (Fig 3C). On the contrary, the transition between the eastern and northern coastal areas and the offshore areas is marked. The latter exhibits a steep slope, reflecting an important change in species composition. Overall, the slope map revealed a high beta diversity gradient in the Tunisian EEZ.

**Fig 4:**
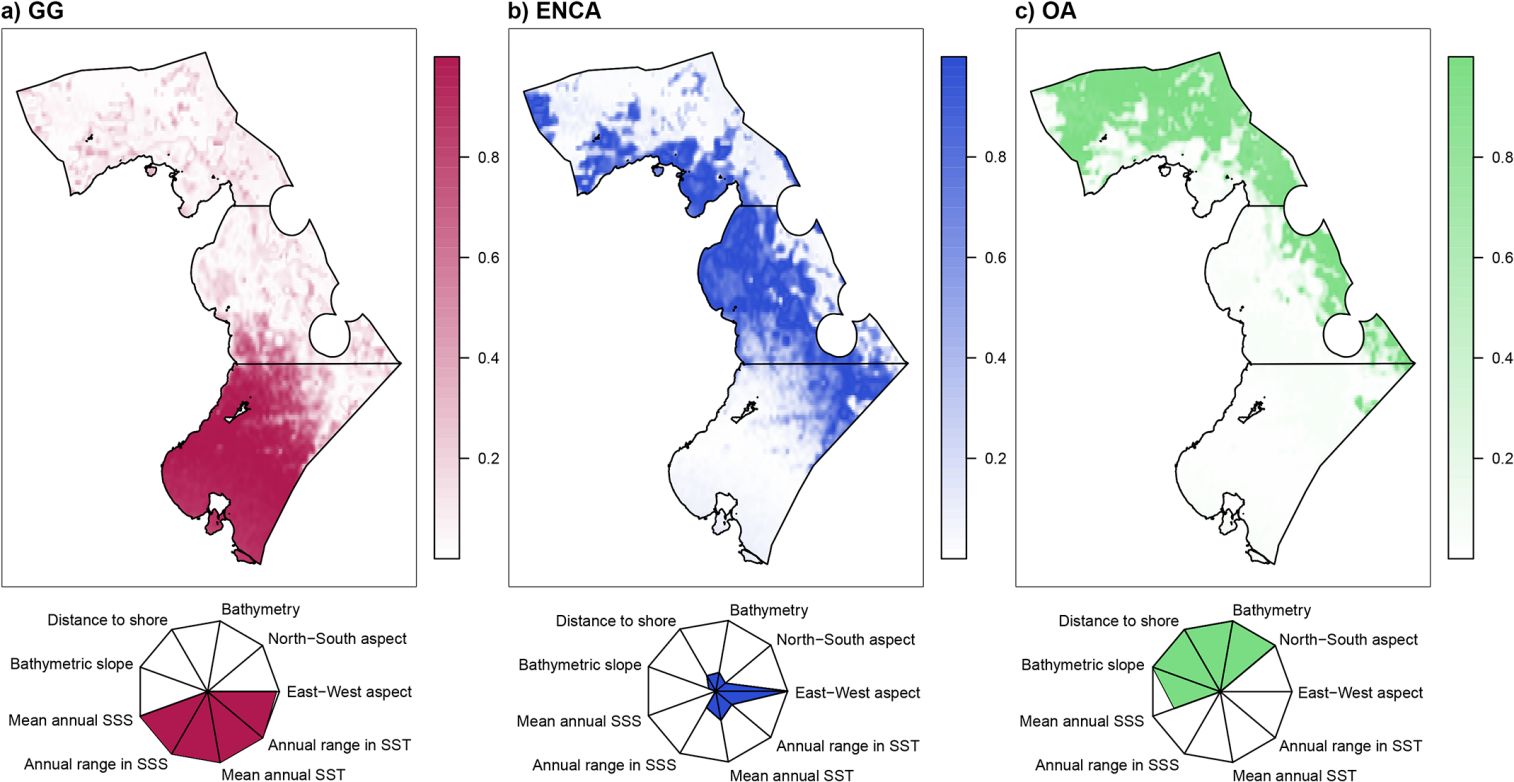
Biogeographical regions resulting from a Fuzzy C means cluster analysis and radial plots of the environmental variables. Radial plots result from the multiplication of the fuzzy membership of each region by the value of each variable (GG: Gulf of Gabes, ENCA: Eastern and Northern Coastal Areas, OA: Offshore Areas).

The three fuzzy biogeographical regions identified using the FCM were each assigned a membership value (Fig 4): (1) southern coastal region (corresponding to the Gulf of Gabes; GG), (2) the eastern and northern coastal areas (ENCA), and (3) the offshore areas (OA). This last region extends further seawards in the north, beyond the continental shelf.

We also generated radial plots to characterize the modeled regions by multiplying the fuzzy membership of each region by the value of each variable.

As this study was predictive, we intentionally omitted any exploration of the factors that may be driving beta diversity. We only relied on the description of the radial plots characterizing each region.

### Indicator species

In total, 14 of the 174 species analyzed had an IndVal^g^ greater than 0.6 (Table 2; from the total 174 species analyzed) and are thus considered to be indicator species.

**Table 2:** Indicator species identified for each biogeographical region on the basis of their indicator values (IndVal^g^).

Biogeographical region	Indicator species	IndVal^g^	p value
Gulf of Gabes (GG)	*Penaeus kerathurus*	0.752	0.001
	*Sepia officinalis*	0.697	0.001
	*Boops boops*	0.61	0.001
Eastern and Northern Coastal Areas (ENCA)	*Octopus vulgaris*	0.694	0.002
	*Mullus surmuletus*	0.648	0.005
	*Loligo vulgaris*	0.644	0.003
	*Mullus barbatus*	0.638	0.001
Offshore Areas (OA)	*Parapenaeus longirostris*	0.733	0.001
	*Lepidopus caudatus*	0.713	0.001
	*Helicolenus dactylopterus*	0.692	0.001
	*Capros aper*	0.683	0.001
	*Merluccius merluccius*	0.667	0.001
	*Nephrops norvegicus*	0.635	0.001
	*Phycis blennoides*	0.6	0.001

## Discussion

It is widely acknowledged that determining beta diversity patterns is a key component for conservation planning and biodiversity management [8,47]. To date, although some Mediterranean basin-scale studies have been completed (e.g., [48]), no efforts have been made to identify the large-scale biodiversity patterns (especially beta diversity) along the Tunisian coast. Indeed, according to the Marine Ecoregions of the World (MEOW) system proposed by Spalding *et al*. (2007) [4], the Mediterranean Sea province is part of the temperate North Atlantic realm and is itself made up of seven ecoregions among which the Tunisian Plateau/Gulf of Sidra. According to the MEOW system, Tunisian EEZ belongs to a unique ecoregion. This system is based on a synthesis of existing biogeographical boundaries and expert knowledge at a global scale.

According to Hattab *et al*. (2015) [49], based on phylogenetic and compositional beta diversity, Tunisian waters belongs to two biogeographical regions, the southern inshore and the southern offshore Mediterranean. This work, carried at the regional Mediterranean scale, used data that were originally sourced from the Atlas of Fishes of the Northern Atlantic and Mediterranean [50] and further refined using the known bathymetric tolerances of species [48].

Neither Spalding *et al*. (2007) [4] nor Hattab *et al*. (2015) [49] take into account local characteristics and are consequently irrelevant to base conservation assessments in the Tunisian EEZ.

As Tunisian authorities are recently committed to adopting an Ecosystem Based Management approach in order to achieve the sustainability of marine ecosystems, there is a need to rest on ecological relevant geographical framework.

### Biogeographical regions delineation

In this study, we showed that for any site within the Tunisian EEZ, the demersal community may exhibit the characteristics of multiple different communities. Further, the degree to which that community belongs to a single community archetype can be quantified on a continuous scale.

The three fuzzy communities delineated in this study reflect the magnitude of dissimilarity in the composition of demersal communities across the Tunisian EEZ. The species assemblages present in the GG biogeographical region are typically characterized by coastal species that favor soft bottom habitats. This region supports the unique Gulf of Gabes ecosystem, which is characterized by the second widest shelf region in the Mediterranean Sea, exclusively soft sediment seafloor habitats (sandy and sandy-muddy) and well-developed *Posidonia* seagrass meadows [24]. The oceanographic conditions of this ecosystem are also unusual, including high tidal activity with up to 2 m tides. Water circulation is cyclonic and localized. Moreover, due to the shallowness of the region, it is highly sensitive to the effects of differential heat [51]. The radial plot indicates that this region is characterized by high SSS (mean annual SSS = 37.46 psu) and SST (mean annual SST = 20.77°C) relative to the other two regions (Fig 4A). Collectively, this unique set of oceanographic and geomorphological characteristics shapes a notable assemblage of species. We identified *Penaeus kerathurus*, *Sepia officinalis* and *Boops boops* to be the indicator species. This finding is consistent with the fact that these are the three most exploited species in the Gulf of Gabes fisheries [20] and that they prefer soft bottom habitats of less than 100 m [52].

The second biogeographical region, ENCA, experiences oceanographic conditions influenced by a stream of the Atlantic Current [51] and is characterized by patchy, diverse seafloor habitats [24]. This region is relatively deep (average depth = 225 m), but experiences lower SSS and SST (mean annual SSS = 37.35 psu and mean annual SST = 20°C) than the GG region (Fig 4B). We identified *Octopus vulgaris* and *Mullus surmuletus* as indicator species of this region. Both species are known to prefer moderate depths and occur in both soft and rocky bottoms [52].

The third biogeographical region, OA, is characterized by very different depth (average depth = 632 m) and exposure conditions as compared to the other two regions (Fig 4C). In addition, its water properties and currents are also very different. This area is flowed by the main stream of the Atlantic Current characterized by cold water with low salinity [53]. Unsurprisingly, the area hosts a very dissimilar assemblage of species. The indicator species we identified for the region include *Helicolenus dactylopterus* and *Lepidopus caudatus*. Both species are typically considered deep-water species that are known to occur at depths of 600m and 620m, respectively [52].

Our results are not consistent with Hattab *et al*. (2015) [49] where Tunisian waters appear to belong to two ecoregions, the southern inshore Mediterranean including the very shallow part of the Gulf of Gabes and the southern offshore Mediterranean including all the remaining EEZ. A biogeographical region including the shallow part of the Gulf of Gabes would have appeared if we hadn't constrained the ordination to three regions to match the number of fisheries management entities. Moreover, as beta diversity depends on the spatial scale, it is unlikely that the three biogegraphical regions delineated at a small scale match with those delineated at a regional scale [54].

This work indicates that both oceanographic and environmental gradients play a role in shaping community composition. However, it is important to highlight that the beta diversity patterns shown here are not only explained by these variables but can also be influenced by a range of other factors that we have not actively considered in this study, such as species interactions [48], bio-physical indicators (e.g. Ekman pumping, nutrient concentration, euphotic depth, stratification) [55] and mesoscale ocean features (e.g. eddy kinetic energy, finite-size Lyapunov exponents, surface frontal gradients) [56]. Moreover, due to the general lack of information available about Tunisian seafloor habitat, we were unable to include habitat type as an explanatory variable. In the future, it is imperative that we focus our efforts on addressing these data limitations to ensure that we can deliver more reliable estimates of beta diversity patterns.

### Implications for conservation and fisheries management

Ecosystem Based Management approach requires a geographical framework for marine zoning [57] and fisheries management [58]. This highlights the need of an objective spatial partitioning of the areas of interest.

Hence, the ability to spatially categorize biodiversity is critical for fisheries managers operating at all scales. In Tunisia, the current fisheries management approach divides national waters into three zones (Fig 1), each of which corresponds to a different administrative entity. These zones are also used by the General Fisheries Commission for the Mediterranean for stock assessment purposes as well as by the INSTM to direct scientific surveys. This zoning regime was adopted in 1994, immediately after Tunisia’s fisheries legislation was amended. These laws were changed to ensure aquatic living resources were sustainably exploited while still preserving the ecosystem. However, the current zoning regime is based on practical and political considerations and is not ecologically relevant. Thus, it is imperative that Tunisia prioritizes the adjustment of these boundaries so that they better reflect the natural ecological boundaries [59,60]. The biogeographical regions identified in this study present one such zoning alternative that could be pursued to achieve this objective.

Further, the beta diversity patterns identified in this study could be used to gauge overall meta-community stability, allowing fisheries managers to better understand the spatial impacts of exploitation and identify recovery opportunities. Indeed, Shackell *et al*. (2012) [61] demonstrated that a low beta diversity scenario can be advantageous when a system is exploited because locally depleted populations are more likely to be "rescued" by neighboring areas. The high levels of beta diversity observed in this study reflect the high variability which characterizes the current Tunisian commercial fishery. This fishery is best defined as a composite fishery, made up of numerous small- to medium-size boats that focus on the coastal region, and a semi-industrial fleet that targets offshore regions. These fishers target multiple species and use multiple gear types. This variability across the fishery makes it difficult to effectively manage [62,63].

The results of this study are also relevant for identifying current data gaps and have the potential to inform future marine surveys undertaken by INSTM. Current surveys neglect two areas in particular: the 0–20 m depth contour off the Kerkenah Islands and the upper EEZ limit, which corresponds to the deepest areas of the continental shelf and the beginning of the continental slope. From our results, we know that the Kerkenah Island region lies within the transition zone between the ENCA and GG biogeographical regions. Thus, it is important that we gather further information about this area as it is likely to be rich in transitional communities of particular ecological interest. The importance of the second area lies in the growing pressures exerted by commercial trawlers as they move to progressively deeper waters as coastal stocks decrease. It is critical that we have the necessary scientific information available to adequately assess these fragile low resilient deep-sea stocks [64]. In contrast, the beta diversity maps could be used to indicate regions that require less sampling effort, thereby improving sampling efficiency. For example, sampling efforts could be reduced in areas identified as having low beta diversity but increased in areas of high beta diversity where there was a greater likelihood of finding rare species and/or rich transitional communities that support key ecological functions [19].

The beta diversity maps could also have direct application for conservation planning. For example, they could be used to assess the efficiency of the current marine protected area (MPA) network and to inform future improvements (i.e., identify high beta diversity areas that can be prioritized for inclusion). This is highly relevant for Tunisia where existing MPAs were developed using the classical alpha diversity indices and are further curtailed by factors such as avoidance of military zones. Unfortunately, these factors have resulted in poorly-located MPAs. For example, although the La Galite and the Zembra archipelago MPAs (Fig 1) may contain important biodiversity, it is clear that their zoning is sub-optimal. Further, as they both occur within a single biogeographical location (i.e., ENCA), they are failing to achieve the biogeographical representation goals widely promoted as best MPA practice (20%–50% coverage by the MPA network) [3]. Thus, it is clear that there is significant opportunity to improve Tunisia’s current MPA network.

Furthermore, it would be relevant to project spatial patterns of beta diversity based on future scenarios of SST and SSS. Since conservation planning requires considering both actual and future patterns, such projections would be relevant to explore.

In conclusion, this study offers the first contribution towards developing a bioregionalisation of Tunisian waters. It provides a basis for future assessments of conservation measures and improvements to fisheries management and conservation planning measures. We recommend that future efforts should focus on exploring the role that environmental variables take in shaping contemporary patterns of beta diversity and the predicted patterns of functional and phylogenetic beta diversities.

## Supporting Information

S1 Fig Monthly frequency of the samplings per geographical region. Data are available for the whole year except for January and November because of meteorological constraints.
(TIFF)

S2 Fig Plot of temperature at sea floor against sea surface temperature (a) and plot of pairwise differences in temperature at sea floor against pairwise differences in sea surface temperature (b). Temperatures originate from MEDAR/MEDATLAS dataset. Temperature at sea floor (TSF) and sea surface temperature (SST) are highly and significantly correlated (p-value < 2.2e-16). The use of SST instead of TSF to predict demersal species assemblages is therefore possible.
(TIFF)

S1 Table List of species considered in this study. (DOCX)
