# Correction: Modeling of Beta Diversity in Tunisian Waters: Predictions Using Generalized Dissimilarity Modeling and Bioregionalisation Using Fuzzy Clustering

**DOI:** 10.1371/journal.pone.0135853

**Published:** 2015-08-19

**Authors:** Frida Ben Rais Lasram, Tarek Hattab, Ghassen Halouani, Mohamed Salah Romdhane, François Le Loc'h

The first author’s name is incorrect in the citation. The correct name is: Ben Rais Lasram F. The correct citation is: Ben Rais Lasram F, Hattab T, Halouani G, Romdhane MS, Le Loc'h F (2015) Modeling of Beta Diversity in Tunisian Waters: Predictions Using Generalized Dissimilarity Modeling and Bioregionalisation Using Fuzzy Clustering.

## References

[1] Lasram FBR, Hattab T, Halouani G, Romdhane MS, Le Loc'h F (2015) Modeling of Beta Diversity in Tunisian Waters: Predictions Using Generalized Dissimilarity Modeling and Bioregionalisation Using Fuzzy Clustering. PLoS ONE 10(7): e0131728 doi:10.1371/journal.pone.0131728 26147371 10.1371/journal.pone.0131728 PMC4492941

