# Supplementary figures and images for: Modeling of Beta Diversity in Tunisian Waters: Predictions Using Generalized Dissimilarity Modeling and Bioregionalisation Using Fuzzy Clustering
Source: PLoS One. 2015 Jul 6;10(7):e0131728. doi: 10.1371/journal.pone.0131728 (PMC4492941; doi:10.1371/journal.pone.0131728)

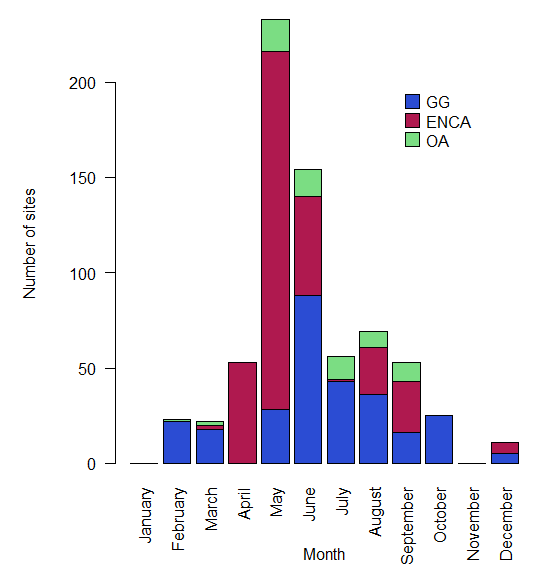

Supplement: S1 Fig — Data are available for the whole year except for January and November because of meteorological constraints. (TIFF) [file pone.0131728.s001.tiff]

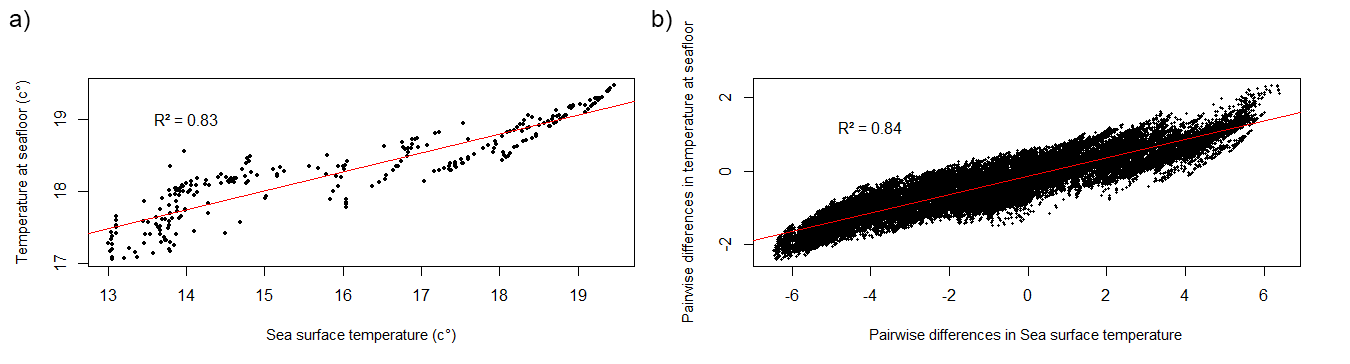

Supplement: S2 Fig — Temperatures originate from MEDAR/MEDATLAS dataset. Temperature at sea floor (TSF) and sea surface temperature (SST) are highly and significantly correlated (p-value < 2.2e-16). The use of SST instead of TSF to predict demersal species assemblages is therefore possible. (TIFF) [file pone.0131728.s002.tiff]
