# Supplementary material for: Modeling of Beta Diversity in Tunisian Waters: Predictions Using Generalized Dissimilarity Modeling and Bioregionalisation Using Fuzzy Clustering
Source: PLoS One. 2015 Jul 6;10(7):e0131728. doi: 10.1371/journal.pone.0131728 (PMC4492941; doi:10.1371/journal.pone.0131728)
Supplement: S1 Table — (DOCX) [file pone.0131728.s003.docx]

| **Ordre** | **Family** | **Genus** | **Species names** |
| --- | --- | --- | --- |
| Anguilliformes | Congridae | Conger | *Conger conger* |
| Aulopiformes | Chlorophthalmidae | Chlorophthalmus | *Chlorophthalmus agassizi bonaparte* |
| Beryciformes | Trachichthyidae | Hoplostethus | *Hoplostethus mediterraneus* |
| Carcharhiniformes | Carcharhinidae | Carcharhinus | *Carcharhinus plumbeus* |
| Carcharhiniformes | Scyliorhinidae | Galeus | *Galeus melastomus* |
| Carcharhiniformes | Triakidae | Mustelus | *Mustelus mustelus* |
| Carcharhiniformes | Triakidae | Mustelus | *Mustelus punctulatus* |
| Carcharhiniformes | Scyliorhinidae | Scyliorhinus | *Scyliorhinus canicula* |
| Carcharhiniformes | Scyliorhinidae | Scyliorhinus | *Scyliorhinus stellaris* |
| Chimaeriformes | Chimaeridae | Chimaera | *Chimaera monstrosa* |
| Decapoda | Alpheidae | Alpheus | *Alpheus glaber* |
| Decapoda | Aristeidae | Aristeus | *Aristeus antennatus* |
| Decapoda | Penaeidae | Metapenaeus | *Metapenaeus Monoceros* |
| Decapoda | Munididae | Munida | *Munida iris* |
| Decapoda | Nephropidae | Nephrops | *Nephrops norvegicus* |
| Decapoda | Palinuridae | Palinurus | *Palinurus elephas* |
| Decapoda | Palinuridae | Palinurus | *Palinurus mauritanicus* |
| Decapoda | Penaeidae | Parapenaeus | *Parapenaeus longirostris* |
| Decapoda | Pasiphaeidae | Pasiphaea | *Pasiphaea sivado* |
| Decapoda | Penaeidae | Penaeus | *Penaeus kerathurus* |
| Decapoda | Palaemonidae | Periclimenes | *Periclimenes granulatus* |
| Decapoda | Pandalidae | Plesionika | *Plesionika acanthonotus* |
| Decapoda | Pandalidae | Plesionika | *Plesionika antigai* |
| Decapoda | Pandalidae | Plesionika | *Plesionika edwardsii* |
| Decapoda | Pandalidae | Plesionika | *Plesionika heterocarpus* |
| Decapoda | Pandalidae | Plesionika | *Plesionika martia* |
| Decapoda | Processidae | Processa | *Processa edulis edulis* |
| Decapoda | Scyllaridae | Scyllarides | *Scyllarides latus* |
| Decapoda | Scyllaridae | Scyllarides | *Scyllarus arctus* |
| Decapoda | Solenocera | Solenoceridae | *Solenocera membranacea* |
| Decapoda | Penaeidae | Trachysalambria | *Trachysalambria curvirostris* |
| Decapoda | Aristeidae | Aristaeomorpha | *Aristaeomorpha foliacea* |
| Gadiformes | Gadidae | Gadiculus | *Gadiculus argenteus* |
| Gadiformes | Gadidae | Gaidropsarus | *Gaidropsarus vulgaris* |
| Gadiformes | Macrouridae | Hymenocephalus | *Hymenocephalus italicus* |
| Gadiformes | Merluciidae | Merluccius | *Merluccius merluccius* |
| Gadiformes | Gadidae | Micromesistius | *Micromesistius poutassou* |
| Gadiformes | Gadidae | Phycis | *Phycis blennoides* |
| Gadiformes | Gadidae | Phycis | *Phycis phycis* |
| Gadiformes | Moridae | Physiculus | *Physiculus dalwigki* |
| Gadiformes | Gadidae | Trisopterus | *Trisopterus minutus capelanus* |
| Gadiformes | Macrouridae | Coelorinchus | *Coelorhynchus coelorhynchus* |
| Hexanchiformes | Hexanchiidae | Heptranchias | *Heptranchias perlo* |
| Lophiiformes | Lophiidae | Lophius | *Lophius budegassa* |
| Lophiiformes | Lophiidae | Lophius | *Lophius piscatorius* |
| Myopsida | Loliginidae | Loligo | *Loligo forbesii* |
| Myopsida | Loliginidae | Loligo | *Loligo vulgaris* |
| Myopsida | Loliginidae | Alloteuthis | *Alloteuthis media* |
| Octopoda | Octopodidae | Eledone | *Eledone cirrhosa* |
| Octopoda | Octopodidae | Eledone | *Eledone moschata* |
| Octopoda | Octopodidae | Octopus | *Octopus vulgaris* |
| Octopoda | Octopodidae | Pteroctopus | *Pteroctopus tetracirrhus* |
| Octopoda | Octopodidae | Scaeurgus | *Scaeurgus unicirrhus* |
| Octopoda | Octopodidae | Macrotritopus | *Macrotritopus defilippi* |
| Oegopsida | Enoploteuthidae | Abralia | *Abralia veranyi* |
| Oegopsida | Ommastrephidae | Todarodes | *Todarodes sagittatus* |
| Oegopsida | Ommastrephidae | Todarodes | *Todaropsis eblanae* |
| Oegopsida | Ommastrephidae | Illex | *Illex coindetii* |
| Osmeriformes | Argentinidae | Glossanodon | *Glossanodon leioglossus* |
| Perciformes | Serranidae | Anthias | *Anthias anthias* |
| Perciformes | Blenniidae | Blennius | *Blennius ocellaris* |
| Perciformes | Sparidae | Boops | *Boops boops* |
| Perciformes | Caproidae | Capros | *Capros aper* |
| Perciformes | Carangidae | Caranx | *Caranx rhonchus* |
| Perciformes | Centracanthidae | Centracanthus | *Centracanthus cirrus* |
| Perciformes | Cepolidae | Cepola | *Cepola macrophthalma* |
| Perciformes | Pomacentridae | Chromis | *Chromis chromis* |
| Perciformes | Sparidae | Dentex | *Dentex dentex* |
| Perciformes | Sparidae | Dentex | *Dentex gibbosus* |
| Perciformes | Sparidae | Dentex | *Dentex macrophthalmus* |
| Perciformes | Sparidae | Dentex | *Dentex maroccanus* |
| Perciformes | Sparidae | Diplodus | *Diplodus annularis* |
| Perciformes | Sparidae | Diplodus | *Diplodus cervinus cervinus* |
| Perciformes | Sparidae | Diplodus | *Diplodus sargus* |
| Perciformes | Sparidae | Diplodus | *Diplodus vulgaris* |
| Perciformes | Serranidae | Epinephelus | *Epinephelus aeneus* |
| Perciformes | Serranidae | Epinephelus | *Epinephelus caninus* |
| Perciformes | Serranidae | Epinephelus | *Epinephelus guaza* |
| Perciformes | Gobiidae | Gobius | *Gobius geniporus* |
| Perciformes | Gobiidae | Gobius | *Gobius niger* |
| Perciformes | Labridae | Labrus | *Labrus viridis* |
| Perciformes | Trichiuridae | Lepidopus | *Lepidopus caudatus* |
| Perciformes | Sparidae | Lithognathus | *Lithognathus mormyrus* |
| Perciformes | Mugilidae | Liza | *Liza aurata* |
| Perciformes | Mugilidae | Mugil | *Mugil cephalus* |
| Perciformes | Mugilidae | Mugil | *Mugil labrosus* |
| Perciformes | Mullidae | Mullus | *Mullus barbatus* |
| Perciformes | Mullidae | Mullus | *Mullus surmuletus* |
| Perciformes | Carangidae | Naucrates | *Naucrates ductor* |
| Perciformes | Sparidae | Pagellus | *Pagellus acarne* |
| Perciformes | Sparidae | Pagellus | *Pagellus bogaraveo* |
| Perciformes | Sparidae | Pagellus | *Pagellus erythrinus* |
| Perciformes | Sparidae | Pagrus | *Pagrus auriga* |
| Perciformes | Sparidae | Pagrus | *Pagrus coeruleostictus* |
| Perciformes | Sparidae | Pagrus | *Pagrus pagrus* |
| Perciformes | Sparidae | Pagrus | *Pagurus cuanensis* |
| Perciformes | Sparidae | Pagrus | *Pagurus prideaux* |
| Perciformes | Haemulidae | Pomadasys | *Pomadasys incisus* |
| Perciformes | Sparidae | Sarpa | *Sarpa salpa* |
| Perciformes | Serranidae | Serranus | *Serranus cabrilla* |
| Perciformes | Serranidae | Serranus | *Serranus hepatus* |
| Perciformes | Serranidae | Serranus | *Serranus scriba* |
| Perciformes | Sparidae | Sparus | *Sparus aurata* |
| Perciformes | Sphyraenidae | Sphyraena | *Sphyraena chrysotaenia* |
| Perciformes | Sphyraenidae | Sphyraena | *Sphyraena sphyraena* |
| Perciformes | Centracanthidae | Spicara | *Spicara flexuosa* |
| Perciformes | Centracanthidae | Spicara | *Spicara maena* |
| Perciformes | Centracanthidae | Spicara | *Spicara smaris* |
| Perciformes | Sparidae | Spondyliosoma | *Spondyliosoma cantharus* |
| Perciformes | Stromateidae | Stromateus | *Stromateus fiatola* |
| Perciformes | Labridae | Symphodus | *Symphodus cinereus* |
| Perciformes | Labridae | Symphodus | *Synchiropus phaeton* |
| Perciformes | Trachinidae | Trachinus | *Trachinus araneus* |
| Perciformes | Trachinidae | Trachinus | *Trachinus draco* |
| Perciformes | Trachinidae | Trachinus | *Trachinus radiatus* |
| Perciformes | Uranoscopidae | Uranoscopus | *Uranoscopus scaber* |
| Pleuronectiformes | Citharidae | Citharus | *Citharus linguatula* |
| Pleuronectiformes | Scophthtalmidae | Lepidorhombus | *Lepidorhombus boscii* |
| Pleuronectiformes | Soleidae | Microchirus | *Microchirus variegatus* |
| Pleuronectiformes | Soleidae | Monochirus | *Monochirus hispidus* |
| Pleuronectiformes | Pleuronectidae | Platichthys | *Platichthys flesus* |
| Pleuronectiformes | Scophthtalmidae | Scophthalmus | *Psetta maxima* |
| Pleuronectiformes | Soleidae | Solea | *Solea aegyptica* |
| Pleuronectiformes | Soleidae | Solea | *Solea lascaris* |
| Pleuronectiformes | Soleidae | Solea | *Solea senegalensis* |
| Pleuronectiformes | Soleidae | Solea | *Solea vulgaris* |
| Rajiformes | Dasyatidae | Dasyatis | *Dasyatis centroura* |
| Rajiformes | Dasyatidae | Dasyatis | *Dasyatis marmorata* |
| Rajiformes | Dasyatidae | Dasyatis | *Dasyatis pastinaca* |
| Rajiformes | Myliobatidae | Myliobatis | *Myliobatis aquila* |
| Rajiformes | Myliobatidae | Pteromylaeus | *Pteromylaeus bovinus* |
| Rajiformes | Rajidae | Raja | *Raja alba* |
| Rajiformes | Rajidae | Raja | *Raja asterias* |
| Rajiformes | Rajidae | Raja | *Raja circularis* |
| Rajiformes | Rajidae | Raja | *Raja clavata* |
| Rajiformes | Rajidae | Raja | *Raja melitensis* |
| Rajiformes | Rajidae | Raja | *Raja miraletus* |
| Rajiformes | Rajidae | Raja | *Raja montagui* |
| Rajiformes | Rajidae | Raja | *Raja oxyrhynchus* |
| Rajiformes | Rajidae | Raja | *Raja radula* |
| Rajiformes | Rhinobatidae | Rhinobatos | *Rhinobatos cemiculus* |
| Rajiformes | Rhinobatidae | Rhinobatos | *Rhinobatos rhinobatos* |
| Scorpaeniformes | Triglidae | Chelidonichthys | *Aspitrigla cuculus* |
| Scorpaeniformes | Sebastidae | Helicolenus | *Helicolenus dactylopterus* |
| Scorpaeniformes | Peristediidae | Peristedion | *Peristedion cataphractum* |
| Scorpaeniformes | Scorpaenidae | Scorpaena | *Scorpaena elongata* |
| Scorpaeniformes | Scorpaenidae | Scorpaena | *Scorpaena maderensis* |
| Scorpaeniformes | Scorpaenidae | Scorpaena | *Scorpaena notata* |
| Scorpaeniformes | Scorpaenidae | Scorpaena | *Scorpaena porcus* |
| Scorpaeniformes | Scorpaenidae | Scorpaena | *Scorpaena scrofa* |
| Scorpaeniformes | Triglidae | Trigla | *Trigla aspira* |
| Scorpaeniformes | Triglidae | Trigla | *Trigla lucerna* |
| Scorpaeniformes | Triglidae | Trigla | *Trigla lyra* |
| Scorpaeniformes | Triglidae | Trigloporus | *Trigloporus lastoviza* |
| Sepiida | Sepiidae | Sepia | *Sepia elegans* |
| Sepiida | Sepiidae | Sepia | *Sepia officinalis* |
| Sepiida | Sepiidae | Sepia | *Sepia orbignyana* |
| Sepiida | Sepiidae | Sepia | *Sepiola affinis* |
| Sepiida | Sepiidae | Sepiola | *Sepiola rondeletii Leach* |
| Squaliformes | Etmopteridae | Etmopterus | *Etmopterus spinax* |
| Squaliformes | Oxynotidae | Oxynotus | *Oxynotus centrina* |
| Squaliformes | Squalidae | Squalus | *Squalus acanthias* |
| Squaliformes | Squalidae | Squalus | *Squalus blainvillei* |
| Stomatopoda | Squillidae | Squilla | *Squilla mantis* |
| Syngnathiformes | Syngnathidae | Hippocampus | *Hippocampus hippocampus* |
| Syngnathiformes | Syngnathidae | Syngnathus | *Syngnathus acus* |
| Syngnathiformes | Syngnathidae | Syngnathus | *Syngnathus phlegon* |
| Syngnathiformes | Centriscidae | Macroramphosus | *Macroramphosus scolopax* |
| Tetraodontiformes | Balistidae | Balistes | *Balistes carolinensis* |
| Tetraodontiformes | Tetraodontidae | Sphoeroides | *Sphoeroides cutaneus* |
| Tetraodontiformes | Monacanthidae | Stephanolepis | *Stephanolepis diaspros* |
| Torpediniformes | Torpedinidae | Torpedo | *Torpedo marmorata* |
| Torpediniformes | Torpedinidae | Torpedo | *Torpedo torpedo* |
| Zeiformes | Zeidae | Zeus | *Zeus faber* |
